# Deterministic Lateral Displacement-Based Separation of Magnetic Beads and Its Applications of Antibody Recognition

**DOI:** 10.3390/s20102846

**Published:** 2020-05-16

**Authors:** Haichao Zhang, Junyi Zeng, Dandan Han, Jinan Deng, Ning Hu, Xiaolin Zheng, Jun Yang

**Affiliations:** Key Laboratory of Biorheological Science and Technology, Ministry of Education, Bioengineering College, Chongqing University, Chongqing 400030, China; haichaozhang2020@163.com (H.Z.); zengjunyier@163.com (J.Z.); 18853607322@163.com (D.H.); biojdeng@cqu.edu.cn (J.D.); huning@cqu.edu.cn (N.H.); zxl@cqu.edu.cn (X.Z.)

**Keywords:** magnetic bead, separation, lateral displacement, microfluidic, antibody

## Abstract

This work presents a magnetic-driven deterministic lateral displacement (m-DLD) microfluidic device. A permanent magnet located at the outlet of the microchannel was used to generate the driving force. Two stages of mirrored round micropillar array were designed for the separation of magnetic beads with three different sizes in turn. The effects of the forcing angle and the inlet width of the micropillar array on the separating efficiency were studied. The m-DLD device with optimal structure parameters shows that the separating efficiencies for the 10 μm, 20 μm and 40 μm magnetic beads are 87%, 89% and 94%, respectively. Furthermore, this m-DLD device was used for antibody recognition and separation among a mixture solution of antibodies. The trajectories of different kinds of magnetic beads coupled with different antigens showed that the m-DLD device could realize a simple and low-cost diagnostic test.

## 1. Introduction

In recent years, deterministic lateral displacement (DLD)-based microfluidic methods have been proposed to effectively separate biological [1,2,3,4] and nonbiological particles [5]. They are passive methods in which particles flow within the fluid through a periodic array of obstacles. The separating efficiency can be improved by optimizing the geometry of the obstacles [6,7,8] and some factors of the viscoelastic fluid [9]. Force-driven DLD (f-DLD), which are driven by external forces, could also achieve the purpose of separating particles of different sizes [10]. Compared to DLD methods, f-DLD methods do not require the precise design of the microstructure or extra pumps. Some external forces, such as centrifugal force [11], electric field force [12,13,14] and gravity [15,16] have been proved to generate f-DLD for the efficient separation of particles of different sizes.

The motion of magnetic beads in microchannels driven by magnetic forces has been widely used in various applications for the manipulation of biological species, such as CTC sorting [17,18,19], DNA extraction [20] and antigen detection [21]. Targets carried by magnetic beads were captured at specific sites under the combined actions of the magnetic force and the fluid for further detections [22]. However, using magnetic force to drive microbeads in a DLD system is rarely reported.

In this study, we designed a novel magnetic-driven DLD (m-DLD) separating device and demonstrated its performance. The magnetic field produced by a permanent magnet was used to drive magnetic beads to flow through a periodic array of cylindrical obstacles. No extra actuating elements such as syringe pumps were required. The effects of forcing angle and inlet width on the separating efficiency of magnetic particles of different sizes were explored. This m-DLD device with optimal parameters was further used as a simple method for the antibody recognition and separation in a mixture solution of antibodies. The trajectories of the magnetic beads carrying different antibodies were analyzed to distinguish the types of antibodies. This m-DLD separating device shows potential as a simple and portable biosensing device for recognizing and separating a mixture of multiple types of target molecules at the same time.

## 2. Materials and Methods

### 2.1. Reagents

Ferroferric oxide (Fe_3_O_4_) magnetic beads (50 mg/mL) with diameters of 10 μm, 20 μm and 40 μm were purchased from Suzhou Zhiyi Microspheres (Suzhou, China). The surface of the bead was modified with carboxyl functional groups for coupling proteins. Alpha-fetoprotein protein (AFP), mouse anti-human AFP, prostate specific antigen (PSA), mouse anti-human PSA, hepatitis B surface antigen (HBsAg) and mouse anti-human HBsAg were purchased from Zhengzhou Cell To Antibody & Antigen Biotech (Zhengzhou, China). FITC-conjugated affinipure goat anti-mouse IgG was purchased from Proteintech Group (Chicago, IL, USA). Sorbitol was purchased from Dingguo Biotechnology (Beijing, China). 4-Morpholineethanesulfonic acid hydrate (MES), 1-ethyl-3-(3’-dimethylaminopropyl) carbodiimide (EDC), N-hydroxysuccinimide (NHS), bovine serum albumin (BSA), and immunostaining fixative were purchased from Shanghai Yuanye Bio-Technology (Shanghai, China).

### 2.2. Microfluidic Chip Design and Fabrication

**Figure 1:**
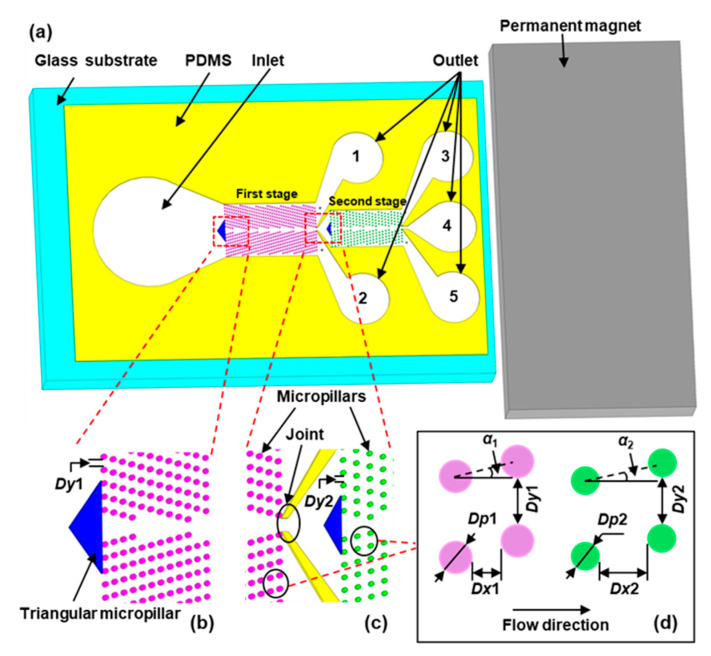
Schematic illustrations of (**a**) the magnetic-driven deterministic lateral displacement (m-DLD) chip, (**b**) the inlet and the first stage of micropillar arrays, (**c**) the junction of the first stage and the second stage and (**d**) parameters of the first and second stages of mirrored cylindrical arrays in the DLD design.

**Figure 2:**
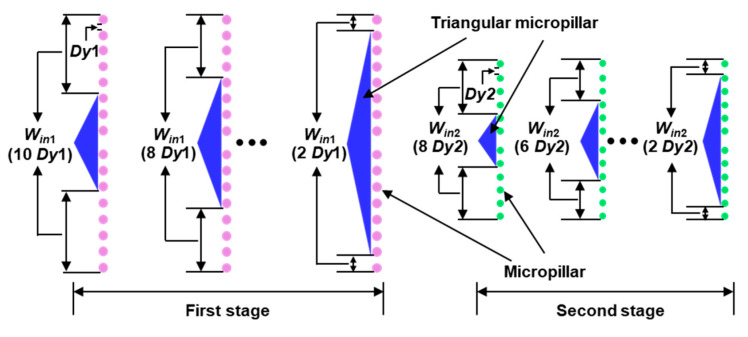
Schematic illustration of different inlet widths for the first and second stages.

The m-DLD chip in this study is 1.2 cm long to ensure that the magnetic force is large enough to drive the motion of magnetic beads, and the number of the micropillar row is enough for a better separating efficiency. One inlet, five outlets (respectively labeled as 1, 2, 3, 4 and 5 in Figure 1a) and two stages of mirrored round micropillars array are fabricated. The middle outlet of the first stage, which is connected to the inlet of the second stage, is defined as the Joint (Figure 1c). At the entrance of each microchannel, a triangular micropillar is designed as a supporting structure and to control the inlet width, allowing particles to enter the microchannel symmetrically, which can help the focusing of flow (Figure 1b). The height of the microchannel is 50 μm. The first-stage micropillar array, consisting of 16 rows and 41 columns of micropillars, is 2.4 mm long and 1.4 mm wide. The length and width of the second-stage micropillar array are 1.9 mm and 0.98 mm, respectively, consisting of 12 rows and 23 columns of micropillars. *Dy*1 and *Dy*2 represent the gaps between the neighboring micropillars in the same row for the first stage and the second stage, respectively (Figure 1b,c). *Dp*1 and *Dp*2 are the diameters of the micropillars in the first and second stages, respectively (Figure 1d). *Dx*1 and *Dx*2 are the distances between the two nearest micropillars in the flow direction in the first and second stages, respectively. In order to achieve a better separating result of the magnetic particles used in this study, we set *Dp*1 = 35 μm, *Dx*1 = 25 μm, *Dy*1 = 50 μm, *Dp*2 = 30 μm, *Dx*2 = 55 μm and *Dy*2 = 50 μm in the experiment according to the reference [23]. The forcing angle is α, which is the angle between the external force and the array of micropillars [24] (Figure 1d). *α*_1_ and *α*_2_ represent the forcing angle of the first stage and second stage, respectively. The inlet width (*W_in_*) is varied by changing the base length of the triangle at the entrance (Figure 2). The effects of the forcing angle and inlet width on the separating results were explored in this study.

For the chip fabrication, a master was formed on a silicon wafer with SU-8 3050 photoresist (Microchem, Newton, MA, USA). Utilizing replica molding method, the pattern on the master was transferred to a polydimethylsiloxane (PDMS) slab. After the treatment of oxygen plasma (PDC-MG, Weike Spectrometer Technology, Chengdu, China), the PDMS slab was bonded at one end of a glass slide to reduce the distance between the magnet and the outlet of the microchannel. On the chip, hole punches were used to form the inlet and outlets, and the inlet diameter (0.4 mm) is larger than the outlet diameter (0.2 mm) to facilitate the loading of the liquid.

### 2.3. Experimental Setup and Method

In order to minimize the effect of the magnetic force along the vertical direction, the microchannel on the chip was aligned with the center line of the magnet (NdFeB N52 permanent magnet, residual magnetic flux density = 3000 Gs, 40 mm × 20 mm × 10 mm), which was used to generate the magnetic force. The rectangular magnet was used to construct the magnetic field, which is conducive to the stable movement of the magnetic beads (see Supplementary Document, Simulation). The magnetic forces at the inlet, Joint and outlet of the chip were measured by a gaussmeter (Weite Magnetic Technology, Shangqiu, China) to be 71.4 mT, 94.4 mT and 126.5 mT, respectively. An aqueous solution containing 60% sorbitol and 0.1% tween-20 was used in the experiment to reduce the Reynolds number of the fluid and prevent the suspended magnetic beads from precipitating on the bottom of the microchannel. The lower density of beads solution (6 × 10^3^–10^4^/mL) was prepared to avoid the agglomeration of magnetic beads. The viscosity and density of the solution were 26.0 mPa·s and 1.293 g/cm^3^, respectively. In order to avoid the influence of the pressure difference between the outlet and the inlet on the movement of the magnetic beads, the solution containing 60% sorbitol and 0.1% tween-20 were introduced into the microchannel by a capillary force before each experiment and stood for 3 min. Then, 1 mL sorbitol/tween-20 aqueous suspension containing 0.01 mg/mL of 10 μm beads, 0.02 mg/mL of 20 μm beads, and 0.04 mg/mL of 40 μm beads was introduced into the microchannel. In order to prevent the magnetic beads from adhering to the microchannel wall at the inlet, the magnet was loaded after all three types of magnetic beads sank to the bottom of the inlet.

The recognition and separation of antibodies were performed by using the m-DLD chip with the optimal structure parameters. The magnetic beads were washed with MES buffer and activated with EDC/NHS. Then 30 μg/mL AFP, PSA and HBsAg were coated on the surface of 10 μm, 20 μm and 40 μm magnetic beads, respectively. Those three types of antigen-coated magnetic beads were added to a mixed solution containing 3 μg/mL mouse anti-human AFP, mouse anti-human PSA and mouse anti-human HBsAg to couple the antibodies. The antibody-coated magnetic beads were then coupled to FITC-conjugated Affinipure Goat Anti-Mouse IgG. The mixture of the as-prepared three kinds of FITC-labeled antibodies carried by magnetic beads was introduced into m-DLD device. The trajectories of magnetic beads were monitored under a fluorescent microscope (IX73, Olympus, Tokyo, Japan). All experiments were repeated at least three times.

## 3. Deterministic Model

**Figure 3:**
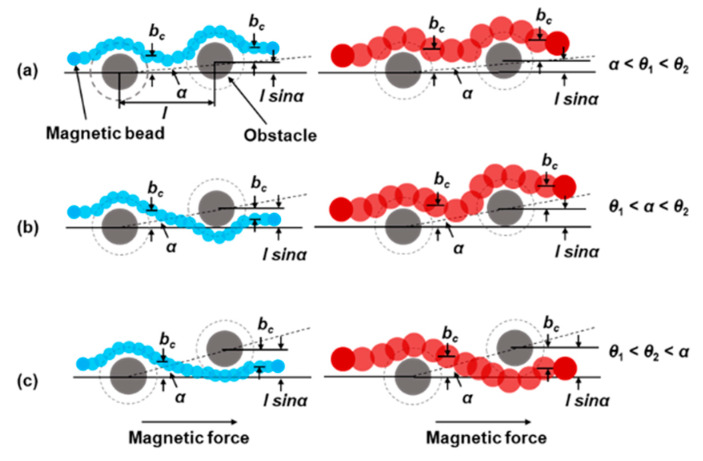
Schematic diagram of the trajectories of particles of two different sizes at different forcing angles. The driving angles are (**a**) *α* < *θ*_1_< *θ*_2_, (**b**) *θ*_1_ < *α* < *θ*_2_ and (**c**) *θ*_1_ < *θ*_2_ < *α*, respectively. We assume that the critical angle (*θ*_2_) of large particles is greater than the critical angle (*θ*_1_) of small particles. The *b_c_* and *l* sin*α* at different forcing angles were given in the schematic diagram.

We use a simple model to describe the trajectory of magnetic beads driven by magnetic force rounding an obstacle (Figure 3). In this model, we assume that the individual particle only collides with the obstacle. The force between particles is neglected. Magnetic force drives the magnetic beads in the horizontal direction.

The critical angle *θ* was defined as the largest forcing angle for which the particles are locked to move along the column of the array [25]. The critical collision parameter is defined as *b_c_*. *l* is defined as the center to center distance between the obstacles. The *b_c_* = *l* sin*α* when the forcing angle *α* is equal to the critical angle *θ*. We show two types of particles in Figure 3, small (blue) ones, critical angles *θ*_1_ and large (red) ones, critical angles *θ*_2_. The critical angle of larger diameter particles is larger than that of smaller diameter particles [26]. So, the *θ*_2_ is assumed to be greater than the *θ*_1_ in the model. Both types of particles move along obstacles for forcing angles below the critical angles *θ*, showing a displacement mode (Figure 3a). As the forcing angle changes to *θ*_1_ < *α* < *θ*_2_ (Figure 3b), larger particles move along the obstacles in a displacement mode, and smaller particles pass the obstacles in a zigzag mode. All particles pass the obstacle and move in a zigzag mode for sufficiently large forcing angles (Figure 3c) [11]. This model indicates that we can adjust the forcing angle to regulate the movement mode of particles to achieve the separation of particles.

## 4. Results and Discussion

Previous reports have shown that the forcing angle and the inlet width are two important parameters that affect the separating efficiency [11]. Therefore, we explored the effects of forcing angle and inlet width in the new designed m-DLD device in this study. The inlet width (*W_in_*) is tuned by changing the base length of the triangular structure, which is defined as a function of *Dy*. For example, *W_in_*_1_ = 2 *Dy*1 means that the inlet width of the first stage contains two gaps between the micropillars. The direction of the magnetic force is fixed, and we varied the forcing angles of the device by fabricating different m-DLD chips with different directions of the micropillar microarrays.

### 4.1. The Influence of the First Stage on the Separating Efficiency

At the first-stage structure, we expect to collect 10 μm magnetic beads at outlets 1 and 2 and allow the 20 μm and 40 μm magnetic beads to flow into the second stage through the Joint. For each kind of magnetic bead, the separating efficiency was defined as the ratio of the number of the beads with a specific size in the desired collection outlet to its total number.

**Figure 4:**
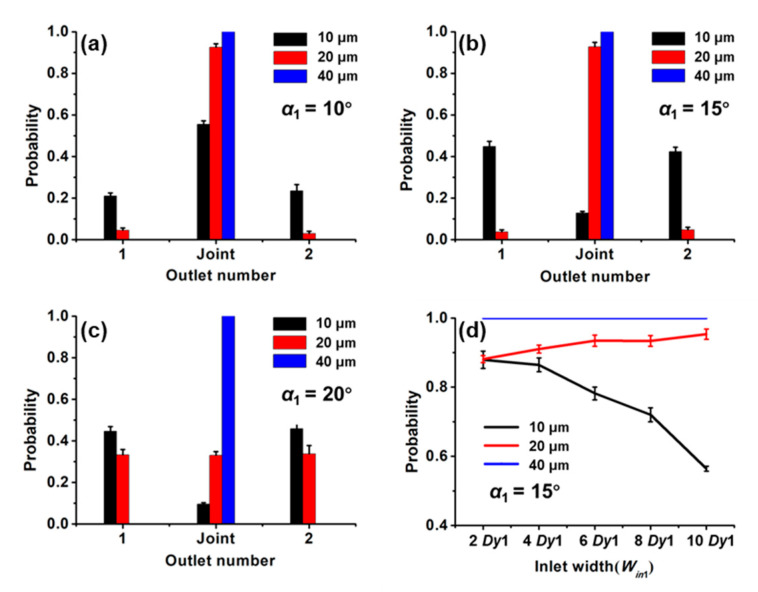
Probability distributions of the magnetic beads at the outlet of 1, Joint and 2 with the forcing angles of (**a**) 10°, (**b**) 15° and (**c**) 20° when the *W_in_*_1_ = 2 *Dy*1. (**d**) Probability distribution of magnetic beads at the outlet of 1, Joint, 2 for different *W_in_*_1_ at *α*_1_ = 15°.

Figure 4a–c shows the effect of the forcing angle on separating effect with the *W_in_*_1_ of 2 *Dy*1. The probability of 40 μm magnetic beads flowing through the Joint is 100%. The variation of *α*_1_ from 10° to 20° does not change the probability of 40 μm magnetic beads in the outlets. This is because the critical angle for 40 μm magnetic beads is larger than the highest forcing angle considered here, and the beads move along the obstacles showing a displacement mode. At the smallest forcing angle (10°) we considered here, the probability of the smallest magnetic bead (10 μm) at the Joint accounts for half of the total number of the smallest magnetic bead in all outlets. As the forcing angle increases to 15°, the probability of the smallest beads (10 μm) at the Joint drops sharply. This is because the forcing angle is larger than the critical angle, and it is in a zigzag mode. At the maximum forcing angle, the 20 μm magnetic beads are almost evenly divided into three outlets because the forcing angle has exceeded the critical angle, and the particles change from the displacement mode to the zigzag mode. It was reported that the critical angle increased with the increase of the diameter of the particles [26], which is consistent with our results. The critical angle for 10 μm beads, 20 μm beads and 40 μm beads are 10°–15°, 15°–20° and >20°, respectively. The excellent sorting efficiency of three kinds of beads was obtained at *α*_1_ = 15° and the sorting efficiency of 10 μm magnetic beads is 87%. The settling velocity of different magnetic beads is different, and some of the magnetic beads have entered the microarray before loading the magnet. This is the main reason for the separation efficiency of the magnetic beads to be less than 100%.

Then, we explored the effect of the inlet width on the separating effect with *α*_1_ = 15° (Figure 4d). With the increase of *W_in_*_1_ from 2 *Dy*1 to 10 *Dy*1, the probability for 10 μm magnetic beads flowing to the outlets 1 and 2 decreased, while the probability of 20 μm and 40 μm magnetic beads flowing through the Joint increased or stabilized. This is a result of the enlarged *W_in_*_1_, which allows the beads entering the micropillars arrays through the central inlets. It increases the probability of magnetic beads flowing toward the Joint due to their moving modes. Therefore, for the optimal separating effect, *α*_1_ = 15° and *W_in_*_1_ = 2 *Dy*1 were selected as design parameters of the first-stage structure.

**Figure 5:**
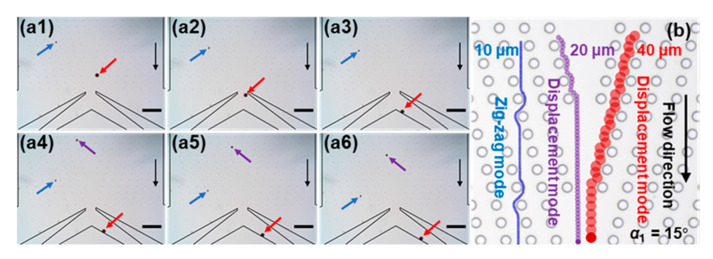
(**a1**–**a6**) Snapshots of the movement of the magnetic beads at the near outlet at the first stage. The time interval is approximately 2.5 s. The 10 μm, 20 μm and 40 μm magnetic beads are indicated by blue, purple and red arrows, respectively. Black arrows indicate the flow direction. Scale bar is 200 μm. (**b**) Schematic illustration of the trajectories of three kinds of magnetic beads at *α*_1_ = 15°.

Figure 5a shows the typical moving mode of three kinds of magnetic beads at the first stage with *α*_1_ = 15° and *W_in_*_1_ = 2 *Dy*1 (see Supplementary Movie, Movie 1). We observed that the larger the diameter of the beads, the faster the moving rate. The 10 μm magnetic bead moves across the micropillars along the direction of the magnetic force and reaches the outlets 1 and 2 (Figure 5a1–a6). The trajectory of 10 μm magnetic bead shows a zigzag mode (Figure 5b). However, the 20 μm and 40 μm beads move along the forcing angle and flow to the Joint (Figure 5a1–a6), indicating a displacement mode (Figure 5b).

### 4.2. The Influence of the Second Stage on the Separating Efficiency

For the second stage, we expect to collect 20 μm magnetic beads at outlets 3 and 5, 40 μm magnetic beads at outlet 4 and no 10 μm magnetic beads at outlets 3, 4 and 5.

**Figure 6:**
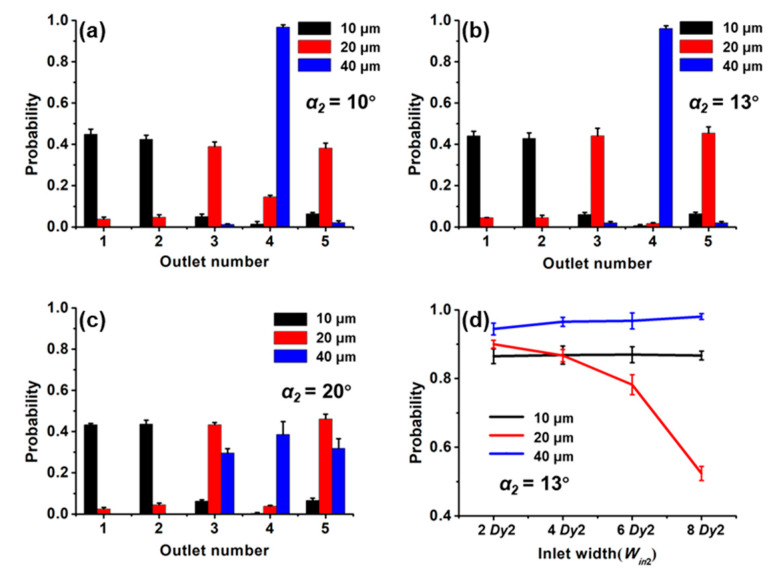
Probability distributions of the magnetic beads at all outlets with the forcing angles of (**a**) 10°, (**b**) 13° and (**c**) 20° when the *W_in_*_2_ = 2 *Dy*2. (**d**) Probability distribution of magnetic beads for different *W_in_*_2_ at *α*_2_ = 13°.

With fixed parameters of the first stage (*α*_1_ = 15°, *W_in_*_1_ = 2 *Dy*1), we explored the effect of different forcing angles on the separating efficiency (Figure 6a–c) with *W_in_*_2_ = 2 *Dy*2. The *α*_2_ hardly affects the distribution of 10 μm magnetic beads at each outlet. The critical angle for the 20 μm and 40 μm magnetic beads are 0°–10° and 13°–20°, respectively. It is different from the critical angles for the two kinds of magnetic beads at the first stage. This is because the critical angle is not only related to the diameter of the particles but also to the parameters of the micropillars. The optimal parameter for the second stage is *α*_2_ = 13°.

Finally, when *α*_1_ = 15°, *W_in_*_1_ = 2 *Dy*1, *α*_2_ = 13°, we explored the effect of the second-stage inlet width on the separating efficiency (Figure 6d). As *W_in_*_2_ increases, the probability of the 20 μm magnetic bead moving to its targeted outlets decreases, while the probability of the 40 μm magnetic bead increases. As the inlet width increases, the probability trend of the magnetic beads moving in the displacement mode is opposite to that of the magnetic beads moving in the zigzag mode, which has been verified at both the first stage and second stage. The optimal separating efficiency of 20 μm and 40 μm magnetic beads with *α*_2_ = 13° and *W_in_*_2_ = 2 *Dy*2 was 89% and 94%, respectively.

**Figure 7:**
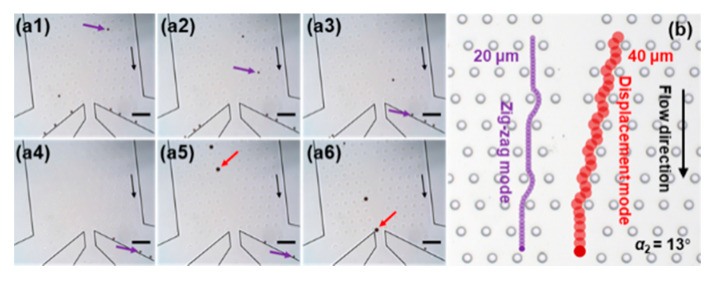
(**a**) Snapshots of the movement of the magnetic beads at the near outlet at the second stage with *α*_2_ = 13° and *W_in_*_2_ = 2 *Dy*2. The time interval is approximately 2 s. The 20 μm and 40 μm magnetic beads are indicated by purple and red arrows, respectively. Black arrows indicate the flow direction. Scale bar is 200 μm. (**b**) Schematic illustration of the trajectories of two kinds of magnetic beads at *α*_2_ = 13°.

Figure 7a shows the typical separating results of the magnetic beads as they approach the outlets in the second stage with *α*_2_ = 13° and *W_in_*_2_ = 2 *Dy*2 (see Supplementary Movie, Movie 2). Figure 7b shows the corresponding trajectories of the two kinds of magnetic beads at *α*_2_ = 13°. The two kinds of magnetic beads have different movement modes and flow to the desired outlets.

### 4.3. Antibody Recognition and Separation

**Figure 8:**
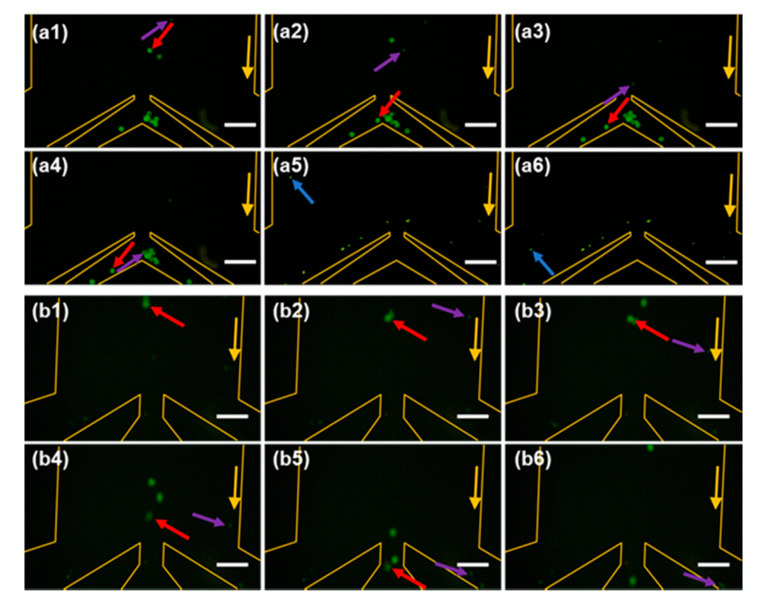
Snapshots of the movement of immunomagnetic beads at the outlet at the first stage (**a1**–**a6**) and the second stage (**b1**–**b6**). The 10 μm, 20 μm and 40 μm magnetic beads are indicated by blue, purple and red arrows respectively. Yellow arrows indicate the flow direction. The time interval is approximately 2 s. Scale bar is 200 μm.

Furthermore, we used the m-DLD device with the optimal structure parameters (*α*_1_ = 15°, *W_in_*_1_ = 2 *Dy*1, *α*_2_ = 13° and *W_in_*_2_ = 2 *Dy*2) to recognize and separate the types of the antibodies. Table 1 illustrates the method to recognize the types of antibodies based on the trajectories of magnetic beads in the first stage and second stage. Figure 8 shows the separating results of a mixture of three types of antibodies near the outlets. We observed two modes, displacement mode and zigzag mode, in both the first stage and second stage. Therefore, we draw a conclusion that the mixture solution contains three kinds of antibodies. Thus, different antibodies can be recognized by their trajectories other than their colors (labels).

## 5. Conclusions

We have demonstrated the feasibility of using magnetic force to separate the magnetic beads by size in a m-DLD device. The application of external force driving DLD separating has been expanded. The effects of forcing angle and inlet width on the separating efficiency in m-DLD device have been investigated. We showed the range of critical angles of the three magnetic beads in the first stage and second stage. We find that as the inlet width increases, the separating efficiency of the magnetic beads in the displacement mode increases and the separating efficiency of the magnetic beads in the zigzag mode decreases. The separating efficiencies for 10 μm, 20 μm and 40 μm magnetic beads are 87%, 89% and 94%, respectively with the optimal parameters of forcing angle and inlet width. We illustrated that the method can be adopted to recognize the types of antibodies based on the trajectories of magnetic beads. With this method, the recognition of multiple of different antibodies in a mixture sample can be realized with only one fluorescent label instead of different labels for each antibody as in common methods.

It is worth noting that this m-DLD-based method can separate and recognize more than three antibodies by increasing the number of stages of the micropillars array. In addition, it was expected to provide a new method to separate cells of the same size through coupling magnetic beads to the cells [27]. Furthermore, by coupling magnetic beads with bacteria, a new method for bacterial classification can also be provided [28].

## Figures and Tables

**Table 1:** Recognition of the types of antibodies based on the trajectories of the magnetic beads.

Trajectories of Magnetic Beads in the First Stage	Trajectories of Magnetic Beads in the Second Stage	Types of Magnetic Beads	Types of Antibodies	Typical Percentage
Zigzag mode	——	10 μm	Mouse anti-human AFP	87%
Displacement mode	Zigzag mode	20 μm	Mouse anti-human PSA	89%
Displacement mode	Displacement mode	40 μm	Mouse anti-human HBsAg	94%
Displacement mode	Zigzag mode, Displacement mode	20 μm, 40 μm	Mouse anti-human PSA, Mouse anti-human HBsAg	89%, 94%
Zigzag mode, Displacement mode	Zigzag mode	10 μm, 20 μm	Mouse anti-human AFP, Mouse anti-human PSA	87%, 89%
Zigzag mode, Displacement mode	Displacement mode	10 μm, 40 μm	Mouse anti-human AFP, Mouse anti-human HBsAg	87%, 94%
Zigzag mode, Displacement mode	Zigzag mode, Displacement mode	10 μm, 20 μm, 40 μm	Mouse anti-human AFP, Mouse anti-human PSA, Mouse anti-human HBsAg	87%, 89%, 94%

## Supplementary Materials

The following are available online at https://www.mdpi.com/1424-8220/20/10/2846/s1, Video S1: Movie 1, Video S2: Movie 2, Simulation.
