# Supplementary material for: Deterministic Lateral Displacement-Based Separation of Magnetic Beads and Its Applications of Antibody Recognition
Source: Sensors (Basel). 2020 May 16;20(10):2846. doi: 10.3390/s20102846 (PMC7287841; doi:10.3390/s20102846)
Supplement: Supplementary file 1 [file sensors-20-02846-s001.zip › sensors-789381-Supplementary Materials/Simulation.pdf]

Simulation:

In the m-DLD system, the main force is the magnetic force, Therefore, we did not conduct the flow simulation. when the cuboid magnet, cylinder magnet, triangular prism magnet, and hexagonal prism magnet were used to generate the magnetic field, we calculated the relationship between the magnetic force and the distance between the bead and the magnet before the experiment, and the simulation model is shown in Figure S1. We set the distance between the magnet and the bead to 0.2 ~ 1.4 cm, and the simulation results are shown in Figure S2. We determined the shape of the magnet used in the experiment based on the simulation result.

In Figure S2, when the size of the magnetic beads is the same, compared with the case where the magnetic beads are in the magnetic field generated by the cylindrical magnet, the triangular prism magnet and the hexagonal prism magnet, the magnetic force generated by the cuboid magnet is the smallest and the magnetic force changes the smallest when the distance changes by the same value. The small change of the magnetic force makes the movement speed of the magnetic bead not change suddenly or greatly in a short time, which is conducive to the stable movement of the magnetic beads. Therefore, considering the stable movement of the magnetic beads, we used the cuboid magnet to construct the magnetic field for experiment.

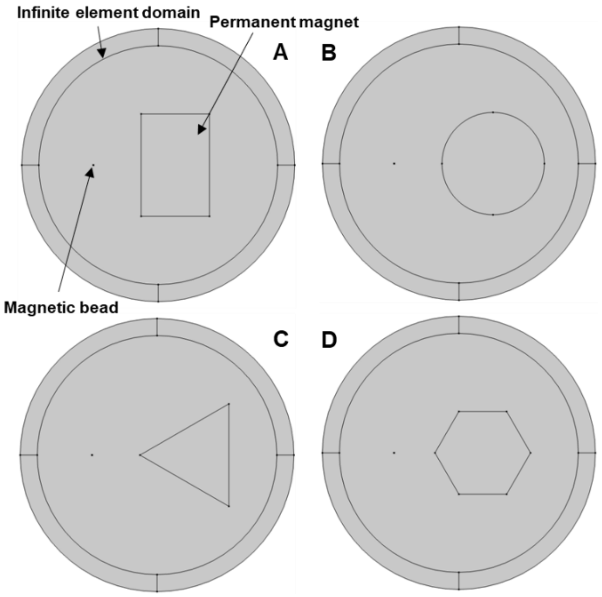

Figure S1. The simulation model of magnet with (A) cuboid, (B) cylinder, (C) triangular prism, (D) hexagonal prism

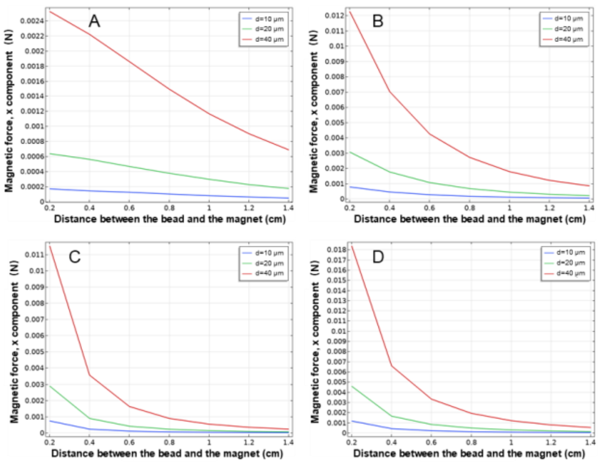

Figure S2. The relationship between the distance and the magnetic force when the shape of the magnet is (A) cuboid, (B) cylinder, (C) triangular prism, (D) hexagonal prism
